# Biorealistic cardiac cell culture platforms with integrated monitoring of extracellular action potentials

**DOI:** 10.1038/srep11067

**Published:** 2015-06-08

**Authors:** Tatiana Trantidou, Cesare M. Terracciano, Dimitrios Kontziampasis, Eleanor J. Humphrey, Themistoklis Prodromakis

**Affiliations:** 1 Nano group, ECS, University of Southampton, Southampton, United Kingdom; 2 Centre for bio-inspired technology, Imperial College London, London, United Kingdom; 3 National Heart and Lung Institute, Imperial College London, London, United Kingdom

## Abstract

Current platforms for *in vitro* drug development utilize confluent, unorganized monolayers of heart cells to study the effect on action potential propagation. However, standard cell cultures are of limited use in cardiac research, as they do not preserve important structural and functional properties of the myocardium. Here we present a method to integrate a scaffolding technology with multi-electrode arrays and deliver a compact, off-the-shelf monitoring platform for growing biomimetic cardiac tissue. Our approach produces anisotropic cultures with conduction velocity (CV) profiles that closer resemble native heart tissue; the fastest impulse propagation is along the long axis of the aligned cardiomyocytes (CVL) and the slowest propagation is perpendicular (CVT), in contrast to standard cultures where action potential propagates isotropically (CVL ≈ CVT). The corresponding anisotropy velocity ratios (CVL/CVT = 1.38 – 2.22) are comparable with values for healthy adult rat ventricles (1.98 – 3.63). The main advantages of this approach are that (i) it provides ultimate pattern control, (ii) it is compatible with automated manufacturing steps and (iii) it is utilized through standard cell culturing protocols. Our platform is compatible with existing read-out equipment and comprises a prompt method for more reliable CV studies.

State-of-the-art technologies to measure conduction velocity (CV) of cell cultures are currently divided into two major groups; (i) optical assays which utilize voltage-sensitive fluorescent dyes and microscopy^1^ and (ii) electrode structures which transduce action potential activity onto electrical signals. Fluorescence has the advantage of recording true intracellular activity, nonetheless, it is toxic to the cells, which significantly limits the lifetime of the culture. Moreover, they are expensive, they are compromised from movement artifacts and they typically show variable emission spectra and fluorescent intensities, known as photobleaching^2^, which can prohibit their reliable use. Planar multi-electrode arrays (pMEAs)^3^,^4^,^5^ have been introduced as a more stable, long-term and minimally invasive recording interface. pMEA technology has been commercialized for 20 years now and has been the state-of-the-art in extracellular field potential measurements of 2D cell cultures^6^,^7^ (Fig. 1a). While the implementation of an MEA can be versatile in terms of substrate and electrode materials and electrode size and layout, the read-out circuitry and stimulation equipment is universal to ensure compatibility with the system. Major laboratory facilities both in pharmaceutical industry and universities have invested in the use of such commercial systems to undertake their research.

Despite the wide applicability of the MEAs as cell culture platforms with integrated monitoring features, currently this technology only supports standard unorganized cell systems. Consequently, these platforms cannot reconstitute the *in vivo* configuration and intercellular interactions. This limitation is particularly prominent in cardiovascular research, because native myocardium has a highly oriented cytoarchitecture. Cardiomyocytes harvested from biopsies and grown *in vitro* typically spread to form an unstructured layer with disorganized myofibrils and diffuse intercellular junctions (Fig. 1b). Myocytes in native myocardial tissue demonstrate an elongated morphology and spatial alignment with intracellular contractile myofibrils oriented parallel to the long axis of each cell and specialized junction complexes (intercalated disks) between adjacent cells located at the ends of each myocyte (Fig. 1c)^8^. The anisotropic structure of cardiomyocytes in native tissue forces the action potential wave to propagate faster in the longitudinal fiber direction. Therefore, CV is higher along the direction of the long axis of the myocytes (longitudinal CV - CVL) and slower in the transverse direction (transverse CV - CVT), indicated by elliptical isochrones as shown in Fig. 1c. As a reference, average CVL in healthy adult rat ventricles is 66–100^9^,^10^,^11^ cm/s with an anisotropy velocity ratio (CVL/CVT) ranging from 1.98 to 3.63^10^,^11^. CV of neonatal rat ventricles is significantly lower than adult, with CVL values ranging within 18.2–21.82 cm/s for two-day old neonatal rats^12^,^13^, however anisotropism is still present. In contrast, action potential in standard unstructured cardiac cultures propagates isotropically (CVL ≈ CVT) (Fig. 1b), with CV of cultured neonatal rat ventricular myocytes (NRVM) being around 23 cm/s^14^,^15^. Clearly, the major limitation of standard cell culture systems is associated with the random spread of excitation, and thus these cell systems cannot account for real tissue behavior and consequently they cannot be used as reliable pharmacodynamic models.

It has been well documented from previous studies that cell patterning determines the extent of alignment and elongation of cardiac myocytes, which further determines the culture CV^14^,^15^,^16^,^17^,^18^,^19^ as well as many other physiological properties of cardiac tissue, such as Ca^2+^ handling^20^,^21^,^22^ and gap junction protein (connexin43) expression^23^. Several different methods have been developed for two dimensional cardiac tissue engineering. The most popular one involves micro-contact printing of extracellular matrix (ECM) molecules on desired locations using elastomeric polymer stamps. The proposed approach is low-cost, easy to use and enables micrometer resolution patterning. Many research efforts have previously engaged commercial pMEAs with micro-contact printing of ECM compounds to achieve guided excitation of cardiomyocytes^16^ and investigate the interactions of a geometrically defined neuronal network^24^. An important limitation of the micro-contact printing method is that alignment of the pattern with the electrode array is hard to control if performed manually. Special equipment (alignment stage) is required in order to ensure precision and reproducibility of the MEA patterning process^16^. In addition, the fabrication process still requires clean room facilities in order to fabricate the masters of the micro-stamps. Furthermore, the elastomeric stamps often transfer contaminants and degrade over time^25^.

Another cell patterning technique involves the selective deposition of ECM proteins and cells on a substrate through microfluidic channels^26^. Polydimethylsiloxane (PDMS) microfluidic channels have been integrated with commercial MEAs to pattern various types of primary neuronal cells into predefined networks^27^. These platforms efficiently enable cell patterning, but the fabrication process is challenging; similarly to the micro-stamping technique, the fabrication involves the manufacturing of Si masters and PDMS mold casting, but additionally it necessitates careful sealing of the PDMS channel network on the MEA surface to prevent leakage during operation. Accurate alignment of the microfluidic channels with the MEA electrodes also requires an alignment stage^27^. Integration of microfluidics and electronics currently comprises a major commercial bottleneck, as existing approaches struggle to make the industrial upscaling financially viable^28^. Furthermore, the application of these hybrid systems in cell culturing is often not straightforward to the end-user, as it usually requires extra equipment, such as syringe pumps.

Micro-abrasion of polyvinyl chloride (PVC) substrates using lapping papers with SiC_4_ crystal heads^14^ or finely brushed collagen-coated glasses^15^ were both previously suggested as effective techniques to produce reusable platforms for uniformly anisotropic cardiac cultures with improved CV. Nonetheless, these techniques cannot be integrated with MEAs, as they are extremely likely to cause local damage to the electrode insulation layer, compromising the overall electrical performance of the MEAs and leading to fractionation of electrograms^29^.

Structured cardiomyocyte cultures have also been realized in three dimensions with the use of naturally or synthetically produced ECMs. Most popular techniques towards this direction involve electrospinning of aligned polymer nanofibers^30^,^31^,^32^ and production of macroporous pre-formed scaffolds with complex biomimetic pore structures^33^. However, integration of these scaffolding technologies with electrical sensors for localized real-time monitoring of cellular activities has not been yet implemented and CV studies have been performed only optically up to date^32^. Preliminary studies demonstrate the potential of integrating nanoelectronics with macroporous 3D scaffolds^34^, yet these approaches are not currently compatible with commercially available recording equipment and they are a long way from commercialization.

Semiconductor manufacturing techniques benefit from the compatibility with automated industrial processes, facilitating maximum resolution, accuracy and reproducibility. Conventional photolithography has been used to generate patterns of self-assembled monolayers (SAMs) and adhesion-promoting molecules, such as poly-D-lysine (PDL) for neural networking^35^ and collagen for NRVM structuring^17^. In a recent study, commercial pMEAs were coated with poly(ethylene glycol) (PEG) silanes which were selectively removed by laser ablation^36^. Fibronectin attached on the PEG-free surfaces, facilitating the confinement of NRVMs in narrow (~20 cells in width) linear pathways to record the effect of pharmacological agents on the CV and refractory period of the cellular network. In another study, custom-made MEAs were lithographically patterned with photoresist and subsequently coated with poly-D-lysine^35^. The MEAs were then rinsed with acetone to remove the remaining photoresist, leaving PDL adsorbed onto specific areas. This method realized neuronal networks of different pattern geometries for electrophysiological studies. When it comes to large-scale production of these platforms, the fabrication process needs to stop after the development of the photoresist, leaving the end-user to coat the MEAs with PDL, rinse with acetone and subsequently seed the cells. A major concern regarding this approach rises from the storage lifetime of the produced platforms, which is determined by the long-term stability of the photoresist pattern, which further depends on the storing conditions and environment.

Obviously the challenges of integrating structuring technologies with monitoring modalities are many; first, the development of scaffolding technologies that are based on biocompatible materials, support long-term and stable patterning and promote a more *in vivo*-like cellular morphology and physiology. Second, the development of monitoring platforms that are compatible with state-of-art well-optimized read-out systems (amplifiers, data acquisition systems, stimulus generators). This will enable the rapid employment of the developed tools in most research facilities, and will bridge the gap between research and commercialization. Finally, the greatest challenge lies in the integration of the two aforementioned features, as it should be time and cost-effective, precisely controlled and reproducible.

We have previously developed a scaffolding technology to structure NRVM on a highly biocompatible material - Parylene C^20^. These polymer scaffolds were produced by selectively modifying Parylene’s inherent hydrophobicity through standard lithography and oxygen plasma, enabling the attachment factors and thus the cells to self-align on the surface of Parylene C-coated glass substrates. The Parylene scaffolds significantly induced cellular alignment and nuclear elongation. Furthermore, the Ca^2+^ cycling of the NRVM was assessed optically and important parameters such as fluorescent amplitude and time to 50% and 90% decay were significantly improved. Here, we leverage this scaffolding technology in combination with commercially available MEAs to deliver an integrated and minimally invasive monitoring platform for electrophysiological studies of biomimetic cardiac tissue. These *application-specific* MEAs are evaluated in terms of: (i) the production of anisotropic cardiac cultures, (ii) electrical performance, (iii) off-the-shelf (storage) lifetime and (iv) reusability.

## Results

### Experimental design

**Figure 1:**
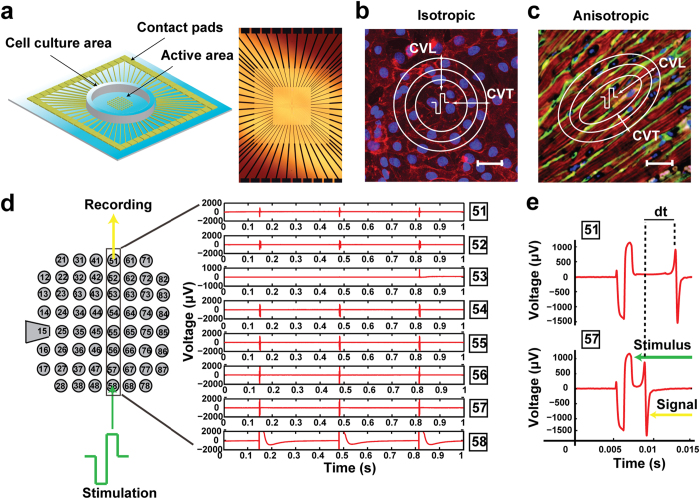
Planar MEA technology for conduction velocity studies. (**a**) pMEA device and close-up of the active area. Structure-function relation in (**b**) isotropic cardiac culture and (**c**) anisotropic cardiac tissue (adapted from 8). Isochrone maps indicate electrical propagation initiated by unipolar point stimulus at center (pulse symbol). CVL and CVT are the longitudinal and transverse velocity. Nuclear DNA and plasma membranes of cardiomyocytes in (b) are stained with DAPI (blue) and di-8-ANEPPS respectively. Adult rat heart in (c) is stained with phalloidin (red), DAPI (blue) and wheatgerm agglutinin (green). Scale bars, 30 μm. (**d**) Example of calculating conduction velocity along column 5: electrode #58 is stimulated and signals from electrodes #57 and #51 are used to calculate activation time. (**e**) Corresponding signals from microelectrodes #57 and #51 are used to calculate difference in activation time (*dt*).

**Figure 2:**
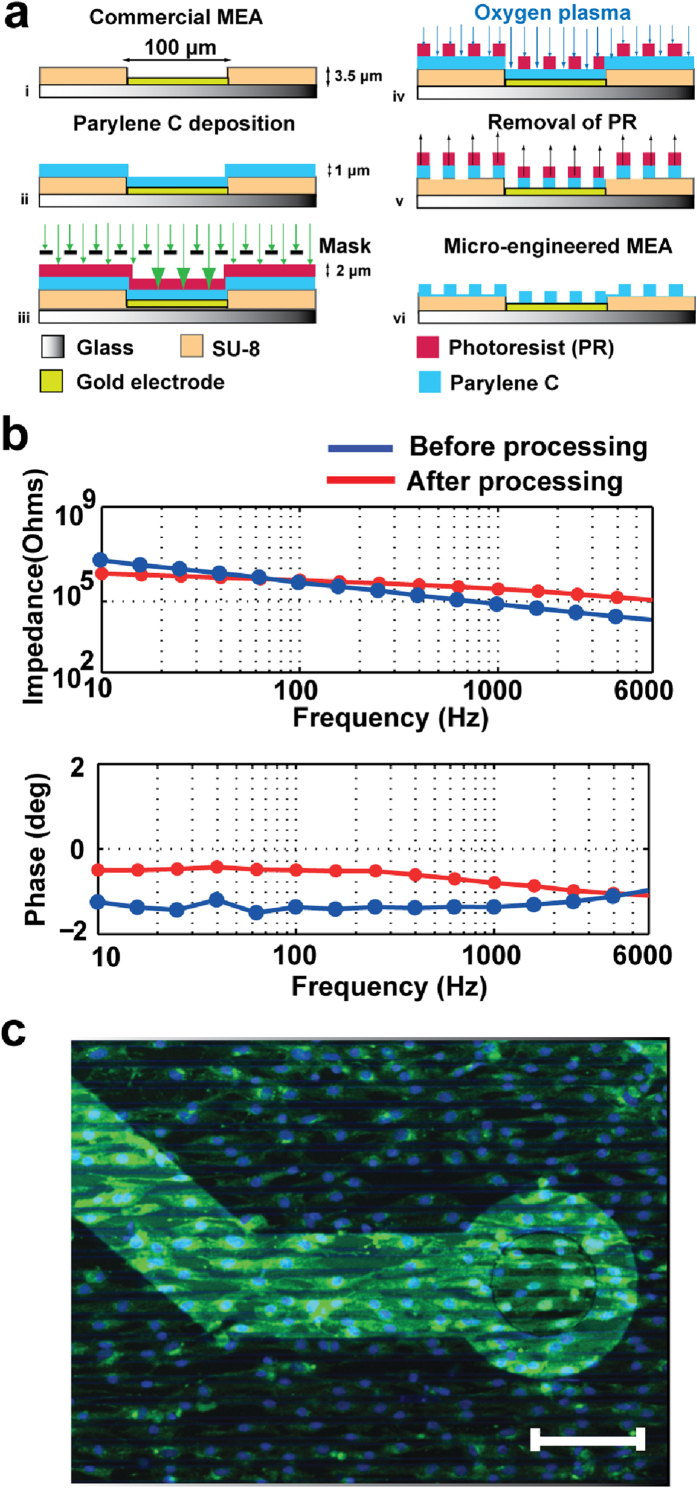
Micro-engineered MEAs. (**a**) Cross-section of the fabrication process. (**b**) Representative impedance measurements versus frequency of a single MEA electrode before (blue) and after (red) processing. (**c**) Immunofluorescence image of NRVM cultured on micro- engineered MEAs stained for plasma membrane (green-wheatgerm agglutinin) and nuclear DNA (blue-DAPI). Scale bar, 100 μm.

Commercially available pMEAs (Multichannel Systems GmbH) were used consisting of a polyimide or glass support substrate with 59 gold circular electrodes (100 μm in diameter and 700 μm interelectrode distance) and an internal gold reference electrode (Supplementary Fig. 1a). Confluent cultures of NRVM were produced on each MEA dish (see Methods). Conduction velocity was calculated along four distinct directions relatively to the internal reference electrode (microelectrode #15); horizontal, vertical, diagonal 45° and diagonal −45°. An electrode at the periphery of the 8 × 8 electrode grid was stimulated with a bipolar pulse and signals from electrodes along the particular direction were measured. Fig. 1d shows the experimental procedure for CV calculation along column 5. Activation time was extrapolated from microelectrodes #57 and #51 to ensure maximum distance and reliability of the calculations (Fig. 1e). Four unprocessed (standard) MEAs served as the control group and six MEAs were micro-engineered to comprise the patterned group. The fabrication process is presented in Fig. 2a and in more detail in the methods section. Representative bright field microscopy images are provided in Supplementary Fig. 1b-d.

### Electrical performance of the micro-engineered MEAs

When an electrogenic cell generates an action potential above an electrode, ions flowing across the cell membrane induce a charge redistribution on the electrode. The frequency of these signals for a cardiomyocyte has been reported to be within a bandwidth of 16 Hz-6 kHz^37^,^38^. Impedance measurements for electrodes before and after processing are presented here within this frequency bandwidth. Fig. 2b demonstrates representative graphs of the impedance modulus and phase of a single electrode before and after the MEA was processed. Typically, manufacturing companies provide impedance references for microelectrodes around an intermediate frequency, usually 1 kHz. According to the manufacturing company these MEAs usually have an impedance of approximately 30 kOhms at this frequency^29^, however, brand new MEAs are often hydrophobic and require a plasma treatment before first use. Therefore, the impedance of the electrodes is initially higher than referenced. Average electrode impedance has increased almost 5-fold after processing (Supplementary Table 1). The introduction of Parylene C areas on top of a metal electrode results both in an extra resistance, which is responsible for an increase in the electrode thermal noise, but also to an extra capacitance. Despite the rise of the impedance levels, recording and stimulation was still possible using bipolar pulses of larger amplitude (2000 – 4000 mV instead of 1400 – 2000 mV peak-to-peak). Based on these measurements, the time delay of the acquired signal can be calculated at 1 kHz according to equation *Δt = φ/360∙f*, where *Δt* is the time shift of the signal, *φ* is the phase angle in degrees and *f* is the frequency of AC voltage applied across the electrode-cell interface. At 1 kHz the average absolute phase angle of the micro-engineered electrodes is 0.8°, and therefore the calculated time delay of the recorded signal is approximately 0.002 ms, which is negligible.

The noise levels of the MEA depend on the electrode size and material, which further depend on the electrode impedance. The smaller the electrode, the higher the impedance and, therefore, the higher are the noise levels. According to the supplier’s specifications, the total maximum noise level for the MEAs employed here and the amplifier is ±10 μV^29^. The baseline noise of micro-engineered electrodes remains within the range ±20 μV (Supplementary Fig. 2). Besides, the magnitude of the acquired electrical signal is such that enables recordings even after introducing Parylene C stripes on top of the gold electrodes.

### Cell morphology and conduction velocity on the micro-engineered MEAs

NRVM seeded on collagen-coated standard MEA dishes did not show any particular alignment (Supplementary Fig. 3a,b). NRVM seeded on collagen-coated micro-engineered MEAs aligned sufficiently to the direction of the lines throughout the electrode grid, as demonstrated in Fig. 2c and Supplementary Fig. 3c,d. Supplementary Fig. 4 demonstrates a close-up of the cells-electrode interface.

**Figure 3:**
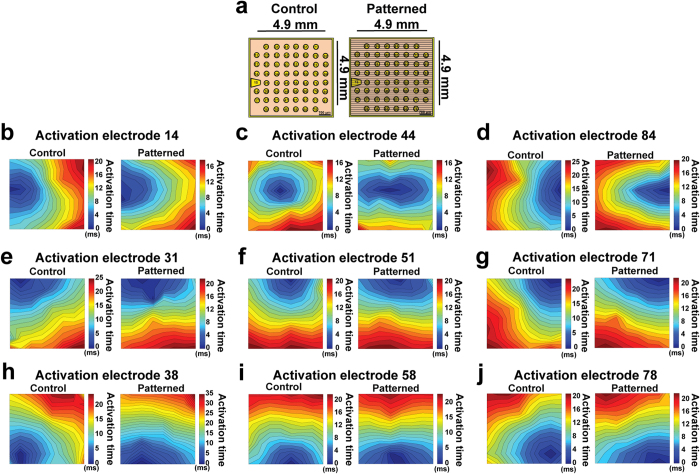
Isochrones of action potential propagation. (**a**) MEA layout and corresponding isochrones of NRVM on unpatterned (control) and patterned MEAs when the culture was stimulated (**b**) from middle left (microelectrode #14), (**c**) from the center (microelectrode #44), (**d**) from middle right (microelectrode #84) (**e**) from top left (microelectrode #31) (**f**) from middle top (microelectrode #51) (**g**) from top right (microelectrode #71) (**h**) from bottom left (microelectrode #38), (**i**) from middle bottom (microelectrode #58) and (**j**) from bottom right (microelectrode #78).

Figure 3 demonstrates representative isochrone maps of NRVM cultures on control and patterned MEAs (Fig. 3a) when stimulated from single electrodes located at nine distinct points at the cell culture area; middle left - electrode #14 (Fig. 3b), middle - electrode #44 (Fig. 3c), middle right - electrode #84 (Fig. 3d), top left - electrode #31 (Fig. 3e), middle top - electrode #51 (Fig. 3f), top right - electrode #71 (Fig. 3g), bottom left - electrode #38 (Fig. 3h), middle bottom - electrode #58 (Fig. 3i) and bottom right - electrode #78 (Fig. 3j). In four analyzed isotropic monolayers the corresponding velocity propagation profile revealed circular isochrones, showing uniform, smooth propagation throughout the culture, which agrees with previously reported studies^14^. Cell elongation and coalignment on the micro-engineered MEAs induced by oriented cell growth was followed by the fastest impulse propagation along the direction of the pattern, resulting in elliptical activation profiles best represented by Fig. 3c.

We also tested the shelf-life of the micro-engineered MEAs by storing them for 60 days before culturing them with NRVM through standard cell culturing protocols. Corresponding conduction velocity studies indicate that the micro- engineered MEAs were still able to promote anisotropic cardiac cultures in terms of cell alignment and CV profiles (Supplementary Fig. 5).

**Figure 4:**
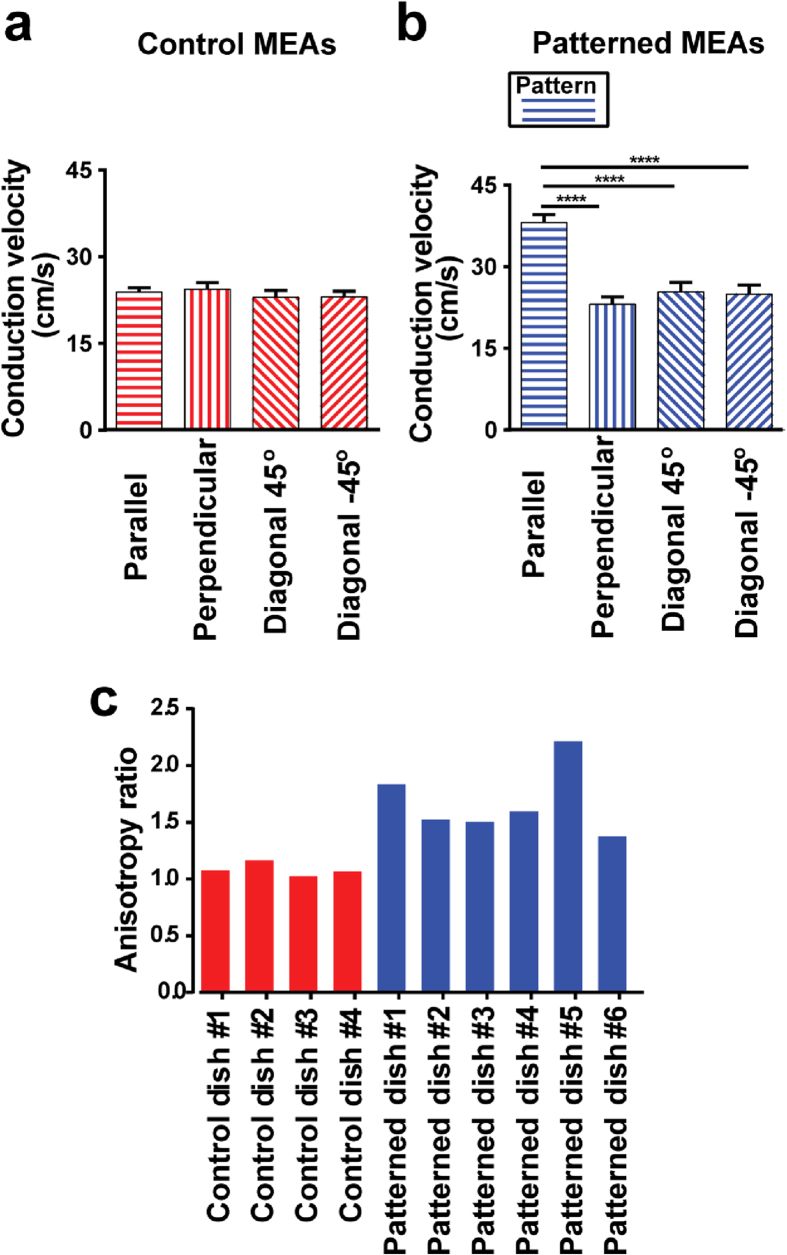
Statistics of conduction velocity study. NRVM on (**a**) unpatterned (*n* = 4) and (**b**) patterned (*n *= 6) MEA dishes. Error bars indicate standard error of means. **** indicate P < 0.0001. Data derived from two isolations. (**c**) Longitudinal-to-transverse velocity anisotropy ratio for control (red) and micro patterned (blue) MEA dishes.

Conduction velocity along the four distinct directions on control MEAs was at similar levels (no statistically significant difference) (Fig. 4a); horizontal 23.87 ± 1.56 cm/s, vertical 24.39 ± 2.21 cm/s, diagonal 45° 22.97 ± 2.39 cm/s and diagonal -45° 23.06 ± 2.00 cm/s. On the contrary, CV along the direction of alignment (CVL) was significantly higher (P  < 0.0001) comparing to all other directions (Fig. 4b); parallel 38.13 ± 3.67 cm/s, perpendicular 23.11 ± 3.30 cm/s, diagonal 45° 25.38 ± 4.24 cm/s and diagonal −45° 25.00 ± 3.99 cm/s. Average longitudinal-to-transverse conduction velocity anisotropy ratio *(r* = CVL/CVT) was significantly (P < 0.05) larger for NRVM on patterned MEA dishes (1.68 ± 0.13) compared to control (1.09 ± 0.03), indicating the production of anisotropic cardiac cultures. Figure 4c presents the *r* values for distinct MEA dishes.

### Reusability of the devices

Our approach is compatible with polyimide-based MEAs, the cost of which is relatively low (£30 per dish) and, therefore, can be used as disposable cell culturing platforms. However, glass-based commercial MEAs are currently expensive (approximately £184 per dish), thus reusing the micro-engineered MEAs is paramount. Reusability of the micro-fabricated platforms will ensure a low-cost and versatile tool for the biologically-oriented researchers, serving as an off-the shelf component for electrophysiological studies. The proposed technique is advantageous, as the MEAs can be recycled after every use; the Parylene membrane is easily peeled-off, the MEAs are cleaned thoroughly according to standard procedures and the fabrication process is repeated. However, here the effectiveness of using the micro-engineered MEAs in two consecutive cultures is validated. By the end of the first experimental week, the MEAs were washed thoroughly with water to remove the cells from their surface (Supplementary Fig. 6). The reusability of the MEAs was quantified in terms of (i) impedance, (ii) cellular alignment and (iii) conduction velocity similarly as previously (see Methods). First, impedance was measured to evaluate the electrical functionality of the electrodes. Subsequently, the MEAs were cultured with NRVM and evaluated for cell alignment and conduction velocity.

**Figure 5:**
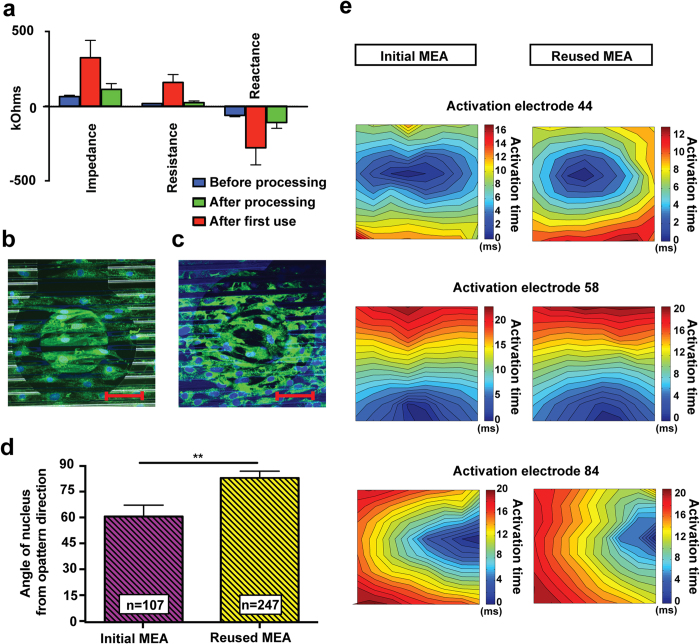
Reusability of micro-engineered MEAs. (**a**) Electrode impedance measurements from a brand-new, non-processed MEA (blue, *n* = 56 electrodes), from the same MEA after processing (red, *n* = 49 electrodes) and after the first use with cells (green, *n* = 56 electrodes). Immunofluorescence images of NRVM cultured on (**b**) initial and (**c**) reused micro- engineered MEAs, stained for plasma membrane (green-wheatgerm agglutinin) and nuclear DNA (blue-DAPI). Scale bars, 50 μm. (**d**) Quantification of cellular alignment. Error bars indicate standard error of mean. *n* represents the number of cells. *** indicate P < 0.001. (**e**) Comparison of conduction velocity profiles of NRVM on an initial (left column) and reused (right column) MEA.

Figure 5a presents average impedance measurements of brand-new MEAs (blue), after they were processed (red) and after their first use with cells (green). The measured electrode impedance is lower than the initial average impedance of the electrodes after processing and before cell culturing. This is most probably attributed to two main reasons; first, the application of voltage biases to electrodes embedded within a thin layer of Parylene C causes a local electrowetting on-dielectric effect, which switches Parylene’s surface from a hydrophobic to a hydrophilic state^39^. This change in hydrophilicity is due to dielectric breakdown of the Parylene layer, which is associated with a rapid reduction in the material’s resistance. Another possible reason for the noticeable decrease in impedance is the fact that the MEAs were maintained in water-based environment (cell medium) for one week during cell culturing, which increases the hydrophilicity of the exposed electrode areas according to the manufacturer^29^, and gradually lowers their impedance.

Cell alignment was quantified using immunofluorescent staining and confocal microscopy. Figure 5b presents a representative fluorescent image from NRVM cultured on a freshly fabricated MEA in comparison with NRVM cultured on the same MEA which was reused for the second time (Fig. 5c). The deviation of the nuclear angles from the direction of the pattern was assessed with appropriate image processing software (see Methods) and results are presented in Fig. 5d. Cells on the reused MEA dishes were less aligned (P < 0.05) than cells cultured on the freshly fabricated MEAs. This result is influenced to a great extent by the presence of unstructured cells sitting on top of the electrodes, which is well-justified by the electrowetting on-dielectric effect previously mentioned. Consequently, there is no distinguishable hydrophilic/hydrophobic pattern on top of the electrodes to selectively absorb the attachment factors and provide guidance for the cells. In the absence of such a pattern, the microtopography itself (~1 μm deep grooves) is not sufficient to force the cells to align. This conclusion is in agreement with studies that employed structure constructs, such as micro-grooved PDMS, where it was reported that a depth of at least 4 μm is essential to achieve sufficient topographical cell patterning^21^. Nonetheless, the majority of cells outside the electrode areas were sufficiently aligned to produce anisotropic cell cultures (*r* = 1.35), exhibiting only small variations in conduction velocity. CV along the pattern direction was still the fastest impulse propagation (32.51 ± 3.18 cm/s) compared to all other three directions, while CV along the transverse direction was still the slowest (24.01 ± 1.50 cm/s). Figure 5e demonstrates representative conduction velocity profiles of the same MEA before and after the first use with cells.

## Discussion

Parylene C is a highly biocompatible material with a long history of use in the encapsulation of implantable microdevices^40^,^41^,^42^. Due to the material’s mechanical robustness and excellent dielectric properties, Parylene is also compatible with existing MEA fabrication flowcharts, commonly employed for insulating the electrodes^42^. The proposed technology is 100% compatible with existing automated silicon manufacturing steps, thus it can be easily adopted by major MEA manufacturing companies (Multichannel systems, Cytocentrics, Qwane Biosciences). Our work also takes into account manufacturing, yield and endurance challenges in exploiting such platforms more widely. For example, the proposed platforms can be stored for at least two months post fabrication without compromising their capability to induce anisotropic cultures. Several existing approaches based on lithographic patterning of adhesion molecules^17^,^35^ require the fabrication and ECM coating to take place one after the other, limiting the storage lifetime of the platforms.

Moreover, the proposed technology provides ultimate control over the pattern production, since it is (i) precise and reproducible, (ii) versatile and (iii) scalable; (i) it relies on conventional well-optimized micro-fabrication processes to allow precise and reproducible pattern layout with minimum deviation, (ii) it can achieve multiple layout geometries tailored to a specific biological application and (iii) it enables microscale patterning resolution down to 1 μm. Other approaches such as micro-stamping and microfluidic patterning, require special consideration when aligning the pattern with the active electrode array, and usually require special equipment (alignment stage) to ensure precise and repeatable patterning of the MEA surface^16^. Our technique can also deliver flexible MEA platforms, since Parylene’s excellent mechanical and thermal properties enable the production of high-density MEAs on free-standing thin (down to 1 μm) flexible films^43^ for a more thorough investigation of the role of the ECM stiffness in cellular function. Finally, the proposed methodology yields MEA platforms that are easy and simple to use, as the ECM compounds (fibronectin or collagen) and cells self-assemble after random plating. This enables the biologically oriented user to efficiently culture these platforms through standard cell culturing protocols.

The electrical functionality of the micro-engineered MEAs was assessed in terms of electrode impedance and noise levels and was found not to affect the performance of the MEAs. Conduction velocity was used to demonstrate the effectiveness of this platform for producing anisotropic cardiac cultures. Anisotropic conduction is determined by the length/width ratio and connectivity of cardiac myocytes, thus it is important for all structuring scaffolds to induce sufficient cell alignment and promote the formation of gap junctions between the cells *in vitro* to facilitate the propagation of the electrical signal across the culture. The anisotropy ratios achieved with our platform (1.38–2.22) were comparable to ratios derived from other models of engineered neonatal cardiac cultures using aligned electrospun fibers (2.0)^32^, microabraded PVC substrates (2.34)^14^ and brushed collagen-coated glass coverslips (1.89)^15^. The average longitudinal conduction velocity of the cells on the micro-engineered MEAs (38.13 cm/s) compared well with those reported for micro-patterned NRVM using a variety of structuring methodologies, such as photolithographic techniques (39 cm/s)^17^, micro-stamping of ECM compounds (36.4 cm/s)^14^, coarse micro-abrasion of glass substrates (37.2 cm/s)^14^, brushed collagen-coated coverslips (34.6 cm/s)^15^ and micro-grooved agar surfaces (30 cm/s)^18^. In addition, average conduction velocity for isotropic monolayers (23.57 ± 2.99 cm/s, *n* = 4 cultures) was intermediate between average values for CVT (23.11 ± 3.30 cm/s, *n* = 6 cultures) and CVL (38.13 ± 3.67 cm/s, *n* = 6 cultures), which is in agreement with previous studies for the same cell type *in vitro*^14^,^44^ and *ex vivo*^12^.

The MEAs were reused in two consecutive cultures and were still able to reliably produce anisotropic cardiac cultures at the expense of a 17.67% on average reduction in the anisotropy ratio, while the electrical functionality of the electrodes was not affected. Considering the high cost of customized MEA platforms and their typical average performance, this error is small and can often be compared with errors produced due to cell culturing protocol variability. Despite this observation, the proposed micro-engineering approach facilitates the recycling of the MEA platforms, as the Parylene film can be easily peeled-off and the fabrication process can be repeated to produce freshly engineered MEAs.

Evaluation of the CV in a cardiac cell culture can reveal important information during drug side effect screening. First of all, a thorough study of the QT interval can shed light on the mechanisms by which drugs cause arrhythmias, and potentially enable the distinction between arrhythmic and anti-arrhythmic effects. Furthermore, a CV investigation can contribute to a better understanding of the pharmacological modulation of cardiac gap junction channels, since the expression and distribution of connexins changes under the presence of certain cardiovascular diseases^45^, which further change the CV of the culture.

Our platforms facilitate the oriented spread of excitation similarly to native heart tissue while they perform minimally invasive and stable electrophysiological monitoring, enabling more reliable CV measurements. The essence of our technology lies in the production of anisotropic cardiac cell cultures based on the self-alignment of the attachment factors and consequently of the cells on a hydrophobic/hydrophilic patterned surface. We thus anticipate that our technology achieves anisotropism independently of the nature of the bathing solution, nonetheless, further research needs to be undertaken to elucidate this. The development of platforms that have high predictive value *in vitro* has significant scientific and commercial impact on drug efficacy and optimization, as it could reduce the cost, time and failure rates of current drug assays^46^. It is hoped that the application of these platforms with cardiac myocytes derived from promising cell lines, such as human induced pluripotent stem cells and embryonic stem cells, can yield human-relevant assays for screening and characterizing novel drug candidates^47^,^48^,^49^,^50^. Our promising initial results encourage the continuation of this work in combination with human cardiomyocytes towards the development of a functional drug screening platform.

## Methods

### Fabrication of the micro-engineered MEAs

Commercially available MEAs (60EcoMEA-w/o and 60EcoMEA-Glass-w/o, Multichannel-Systems GmbH) were purchased comprising an array of 59 circular gold electrodes (100 μm in diameter with 700 μm spacing) and one internal gold reference electrode with SU-8 or polyimide insulated tracks (Supplementary Fig. 1a). The fabrication process is outlined in Fig. 2a. The MEAs were cleaned with acetone (MicroChemicals GmbH), isopropyl alcohol (MicroChemicals GmbH) and deionized (DI) water, blown dry with nitrogen and dehydrated at 90 °C for 90 s. The pads were covered with adhesive tape and the MEAs were subsequently coated with 1 μm of Parylene C (SCS, Cat#980130-C-500GE) by chemical vapor deposition, using a commercially available coater (PDS2010, SCS) by vaporizing (150 °C) and then pyrolizing Parylene C dimer (690 °C). Hexamethyldisilazane (HMDS) (Sigma-Aldrich, Cat#40215-1L) was applied before a thin layer (2 μm) of positive photoresist AZ5214E (MicroChemicals GmbH) was spin-coated on the Parylene-coated MEAs. The samples were then soft baked on a hotplate at 90 °C for 90 s, selectively exposed to UV light for 60 s through a chrome-plated glass mask consisting of transparent areas with parallel 10/10 μm lines. The samples were subsequently developed in AZ400k (MicroChemicals GmbH) (4:1 DI water: AZ400k) for 20 s to remove the exposed photoresist. Parylene was then oxygen plasma etched with an inductively coupled plasma etcher (OPT 100 ICP 380, Oxford Instruments Plasma Technology) at 1000 W plasma source power for 3 min and 20 s at a working pressure of 10 mTorr, 50 sccm O_2_ flow and 0 °C temperature. The etching rate achieved was approximately 380 nm/min. To achieve anisotropic etching of Parylene C and optimize vertical etching, oxygen plasma was performed at a very low pressure (10 mTorr), while oxygen ions were adequately accelerated and directed perpendicular to the surface using a radio frequency power of 20 W, which corresponds to a bias voltage of approximately 100 V between the electrodes. Before inserted into the plasma reactor, samples were prebaked on a hotplate at 110 °C for 90 s for hardening the protective photoresist mask. After plasma etching the remaining photoresist was removed through immersing the MEAs into acetone, isopropyl alcohol and deionized water. A stripped pattern of SU-8/Parylene or polyimide/Parylene was created on top of the insulating tracks and accordingly a pattern of gold/hydrophobic Parylene on top of the electrodes (Supplementary Fig. 1b-d). For the control group standard non-processed MEAs were used.

### Sample preparation and electrode impedance measurements

The electrical functionality of the MEAs before and after processing was assessed through measurements of the impedance of the micro-electrodes. Electrode impedance measurements were performed using an impedance analyzer (COMPACTSTAT, Ivium Technologies) and appropriate software (Soft. rel. 2.2.18, Ivium Technologies). A four-electrode configuration is provided by the system (working, sensing, counter and reference electrode), but in this study a two-electrode configuration was employed, connecting the working with the sensing electrode and the reference with the counter electrode. Before measuring, the MEAs were washed with ethanol and left to dry. Impedance of each electrode was measured in the range of 10 Hz-1 MHz (applying a signal of 10 mV) in conductive solution (phosphate buffer saline) versus an Ag/AgCl bath electrode (CHI11P, IJ Cambria Scientific Ltd). Before performing the impedance scan, the open-circuit potential was recorded to ensure that no current passes between the working and the reference electrode that will polarize the latter. The open-circuit potential is an indication of the chemical composition of the interface and it can be used for quality control of the initial interface conditions. Average open-circuit potential values for electrodes of non-processed and processed MEAs were 0.325 ± 0.054 V and 0.270 ± 0.083 V respectively.

### Cardiomyocyte isolation and culture

NRVM were isolated from Sprague-Dawley rats 2 days after birth using the GentleMACs Neonatal Heart Dissociation Kit (Mi Kit -Miltenyi Biotec GmbH). All experiments were conducted in accordance with Home Office regulations detailed in the Animals (Scientific Procedures) Act 1986. The preparation steps include the reagent preparation step 1 using medium-199 (Corning), the dissociation protocol step 1 using Hank’s balanced salt solution (Sigma-Aldrich, Cat#H6648), the dissociation protocol steps 11–13 using medium-199 supplemented with 10% neonatal calf serum, also 50 U/ml penicillin, 2 mg/ml vitamin B12 and 200 mM L glutamine (all Sigma-Aldrich), the dissociation protocol step 14, centrifugation at 1000 rpm, for 5 min. Subsequently, the solution was resuspended in 20 ml supplemented medium-199 and preplated for 1 h to remove fibroblasts. The cells were then counted using a haemocytometer. Prior to seeding, all MEAs and silicon rings (diameter approximately 6 cm) were sterilized by double submersion in 70% ethanol (Sigma-Aldrich) for 5 min. They were then left to dry in a sterile environment. The silicon rings were sealed on the center of the MEA, which was subsequently washed twice with sterile phosphate buffer saline (Oxoid, Cat#BR0014) and then with sterile deionized water to remove any crystal compounds from the surface. After drying out, the MEAs were coated with 5 mg of type IV human placenta collagen (Sigma-Aldrich, Cat#C7521) diluted in 22 ml of Hank’s balanced salt solution and 3 ml of glacial acid. The solution was applied for 1 min, and was succeeded with 2 quick washes with sterile deionized water. During the last step, the MEAs were immersed into water and stored for 1 h in the incubator (37 °C, 5% CO_2_). Before seeding, water was removed. 50 × 10^3^ cells in 10 μl culture medium (67% Dulbecco’s modified eagle medium (Gibco), 16% medium-199, 10% horse serum (Sigma-Aldrich), 4% fetal bovine serum (Sigma-Aldrich), 2% 1 M HEPES (Sigma-Aldrich) and 1% penicillin-streptomyosin (Sigma-Aldrich) were seeded on each MEA. All MEAs were then incubated for half an hour to allow the cells to attach on their surface. Subsequently, the container plate was gently filled with cell medium (500 μl). Complete medium was changed 18 h after seeding. Before changing the medium, the MEAs were gently shaken for 1 min clockwise and 1 min anticlockwise to detach the dead cells. All experiments were performed at day 4 post-seeding.

### Sample preparation and electrophysiological recordings

Particular attention was paid on the formation of uniformly anisotropic and isotropic cultures, so that cell monolayers had no anatomical discontinuity caused by under-confluence of cells. Before recording, cell cultures were assessed under the microscope and discarded if not fully confluent. Electrophysiological recordings of the cells were performed using a 60 channel pre- and filter amplifier (MEA1060-Inv, Multichannel systems GmbH) and a USB data acquisition system (USB-MEA64-System, Multichannel systems GmbH) at a sampling frequency of 25 kHz. Point-stimulation of cells was realised through application of voltage-driven electrical stimulation through a 2-channel general-purpose stimulus generator (STG2004, Multichannel systems GmbH). Stimulation was achieved using a bipolar voltage pulse with total duration 2 ms and amplitude ranging from 1400-4000 mV peak-to-peak. Data acquisition and setup of the stimulation electrodes were achieved with the use of dedicated software (MC Rack and MC Stimulus II, Multichannel systems GmbH). During recordings, the cell culture was kept in warmed (37.1 °C) cell culture medium. Cultures were equilibrated for at least 5 min, and a 10 s-recording was made in the absence of electrical stimulation to check for spontaneous activity and assess the presence of active cells on the electrodes. A bipolar voltage pulse was used to stimulate each time an electrode at the edge of the electrode grid (electrodes 1x, x1, 8x, x8, 22, 27, 72 and 77). This approach was adopted in order to ensure maximum distance over the signal propagation and increase the reliability of the measurements. Voltage and stimulation frequency were adjusted for each MEA dish according to the spontaneous beating frequency of the culture, so that the cells follow the applied stimulus. Action potentials were recorded from 59 sites.

### Data handling of electrophysiological recordings

The activation time was defined as the instant of maximum negative slope of the action potential. Activation time and corresponding isochrones were produced using software written in MATLAB^®^. Isochrones were produced using the maximum number of available electrodes (at least 50 for each case). In cases where electrodes did not record analyzable signals due to noise, missing contacts or large stimulus artefact, linear propagation was assumed and the activation time of the electrode was calculated through interpolation between the two neighboring electrodes. Knowing the interelectrode distance, conduction velocity between two electrodes on the same direction line was calculated as the interelectrode distance over the difference in activation time of the signals acquired from the two electrodes (Fig. 1d,e). Average conduction velocity for each MEA dish was calculated along four distinct directions relatively to the position of the reference electrode; a) horizontal, b) vertical, c) diagonal (clockwise) at 45° and d) diagonal (anticlockwise) at –45^o^. For horizontal direction measurements, electrodes 1x, 8x, 21, 71, 28, 78 were stimulated one-by-one and the signals were recorded each time along the corresponding lines. For vertical propagation electrodes x1, x8, 12, 17, 82, 87 were stimulated. Figure 1d shows an indicative example of one such measurement to calculate conduction velocity along column 5. Electrode 58 is stimulated and the signals are recorded along this column. Knowing the distance between electrodes 57 and 51 (in this case 4.2 mm), conduction velocity is calculated. To calculate conduction velocity along the diagonal direction (45° to the reference electrode), electrodes 12, 13, 14, 21, 22, 31, 41, 58, 68, 77, 78, 85, 86, 87 were stimulated. Finally, to calculate conduction velocity along the diagonal direction (−45° to the reference electrode), electrodes 16, 17, 27, 28, 38, 48, 61, 71, 72, 82, 83, 84 were stimulated. Average conduction velocity for each direction was calculated for each MEA dish. Statistical analysis of conduction velocity of all MEA dishes was performed with Prism 4 software (GraphPad software Inc) using a one-way ANOVA Tukey test with a confidence interval of 95%.

### Sample preparation and imaging of cardiomyocytes using confocal microscopy

Immunofluorescent staining was used to assess the density and alignment of the cells on the MEAs. The cells were washed thrice in phosphate buffer saline and fixed in ice cold methanol (Sigma-Aldrich) for 5 min. Subsequently, the cells were blocked in 1% bovine serum albumin (Sigma-Aldrich) for 30 min at room temperature. Wheatgerm agglutinin (Sigma-Aldrich) was diluted in 1% bovine serum albumin (1:20). Bovine serum albumin was removed from the cells and wheatgerm agglutinin was added for 45 min at room temperature in the dark. The cells were subsequently washed in phosphate buffer saline three times. Nuclear DNA was stained using 1 μl DAPI (Invitrogen) in 1 ml phosphate buffer saline for 10 min at room temperature in the dark. Finally, the cells were washed thrice in phosphate buffer saline and mounted in Citifluor mountant (Agar scientific) using a large glass coverslip. Cellular alignment was assessed based on the angle of the major elliptic axis of individual nuclei with respect to the direction of the patterning. The nuclear angles were measured using built-in functions of a dedicated image processing software tool (Fiji, NIH). Statistical analysis of cellular alignment was performed with Prism 4 software (GraphPad software Inc) using an unpaired t-test (two-tailed) with a confidence interval of 95%.

### Sample preparation and imaging of cardiomyocytes using scanning electron microscopy

Cells were fixed in 3.7% formaldehyde solution (Sigma Aldrich, Cat#F1635-25ML) in phosphate buffer saline (1:9) for 15 min at room temperature in a fume hood. The cells were then rinsed twice with fresh buffer (no fixative added). Buffer solution was replaced with 50% ethanol in distilled water for 20 min. The process was continued with 60%, 70%, 80%, 90% and 100% ethanol solutions, each left for 20 min. The final step was repeated with 100% ethanol solution left on the cells for another 20 min. After the dehydration procedure was completed, the samples were transferred from 100% ethanol into a 1:2 solution of HMDS: 100% ethanol for 20 min. The samples were then transferred to a fresh solution of 2:1 HMDS: 100% ethanol for 20 min. Finally, the samples were transferred into 100% HMDS for 20 min, and this step was repeated. When the samples were submerged in the final 100% HMDS solution, the MEAs were capped loosely in the fume hood overnight to enable evaporation for the HMDS. The samples were coated with a thin layer of gold using a sputter-coater (IN02, Emitech) at a voltage of 0.8 kV, current of 5 mA and pressure of 6 × 10^−2^ mbar for 30 s. Scanning electron microscopy was performed using an EVO-SEM (EVO LS25, Zeiss) at 15 kV.

## Additional Information

**How to cite this article**: Trantidou, T. *et al.* Biorealistic cardiac cell culture platforms with integrated monitoring of extracellular action potentials. *Sci. Rep.* **5**, 11067; doi: 10.1038/srep11067 (2015).

## Supplementary Material

Supplementary Information
