# Supplementary material for: Biorealistic cardiac cell culture platforms with integrated monitoring of extracellular action potentials
Source: Sci Rep. 2015 Jun 8;5:11067. doi: 10.1038/srep11067 (PMC4459200; doi:10.1038/srep11067)
Supplement: Supplementary Information [file srep11067-s1.pdf]

Supplementary information

Biorealistic cardiac cell culture platforms with integrated monitoring of extracellular action potentials

Tatiana Trantidou, Cesare M Terracciano, Dimitrios Kontziampasis, Eleanor J Humphrey & Themistoklis Prodromakis

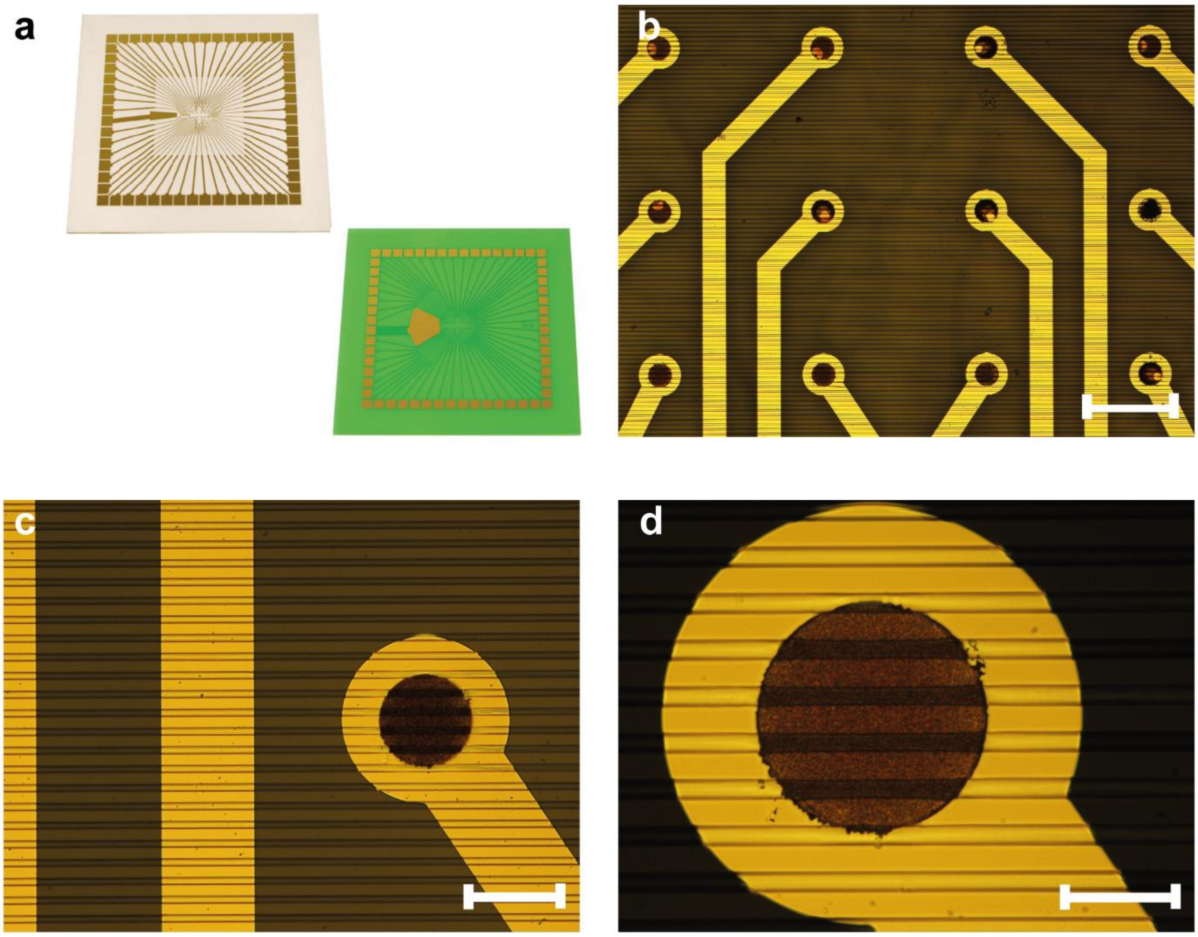

Supplementary Figure 1 | Micro-engineered MEAs. **(a)** Commercially available MEAs used in this study. Bright field microscopy images of micro-engineered MEAs, showing single electrodes at **(b)** x5, **(c)** x20 and **(d)** x50 magnification. Scale bars, 350 µm **(b)**; 100 µm **(c)**; 50 µm **(d)**.

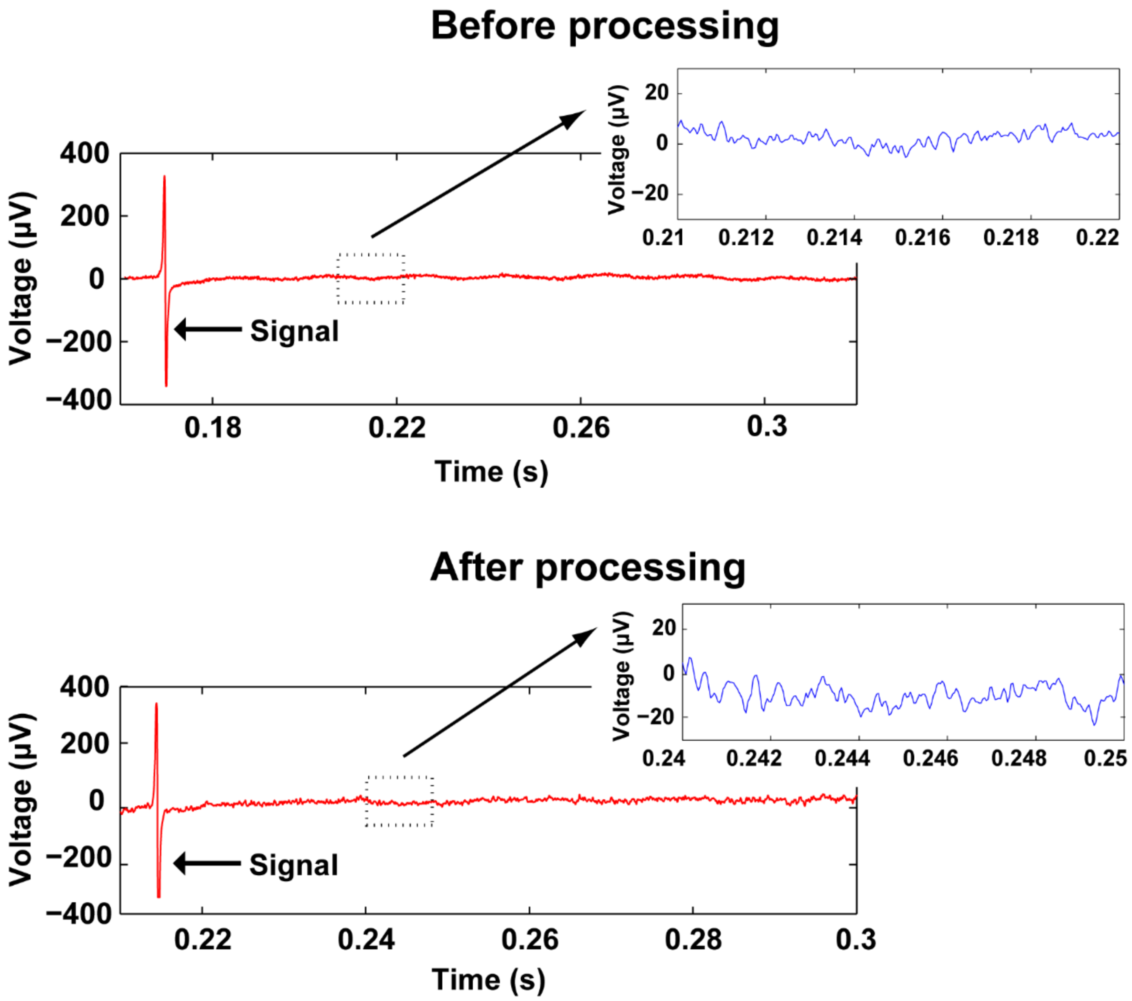

Supplementary Figure 2 | Noise levels of micro-engineered MEAs. Electrical recordings from single electrode before (top) and after (bottom) processing, indicating the signal) generated by NRVM (cut-off at 400 µV. Unfiltered data.

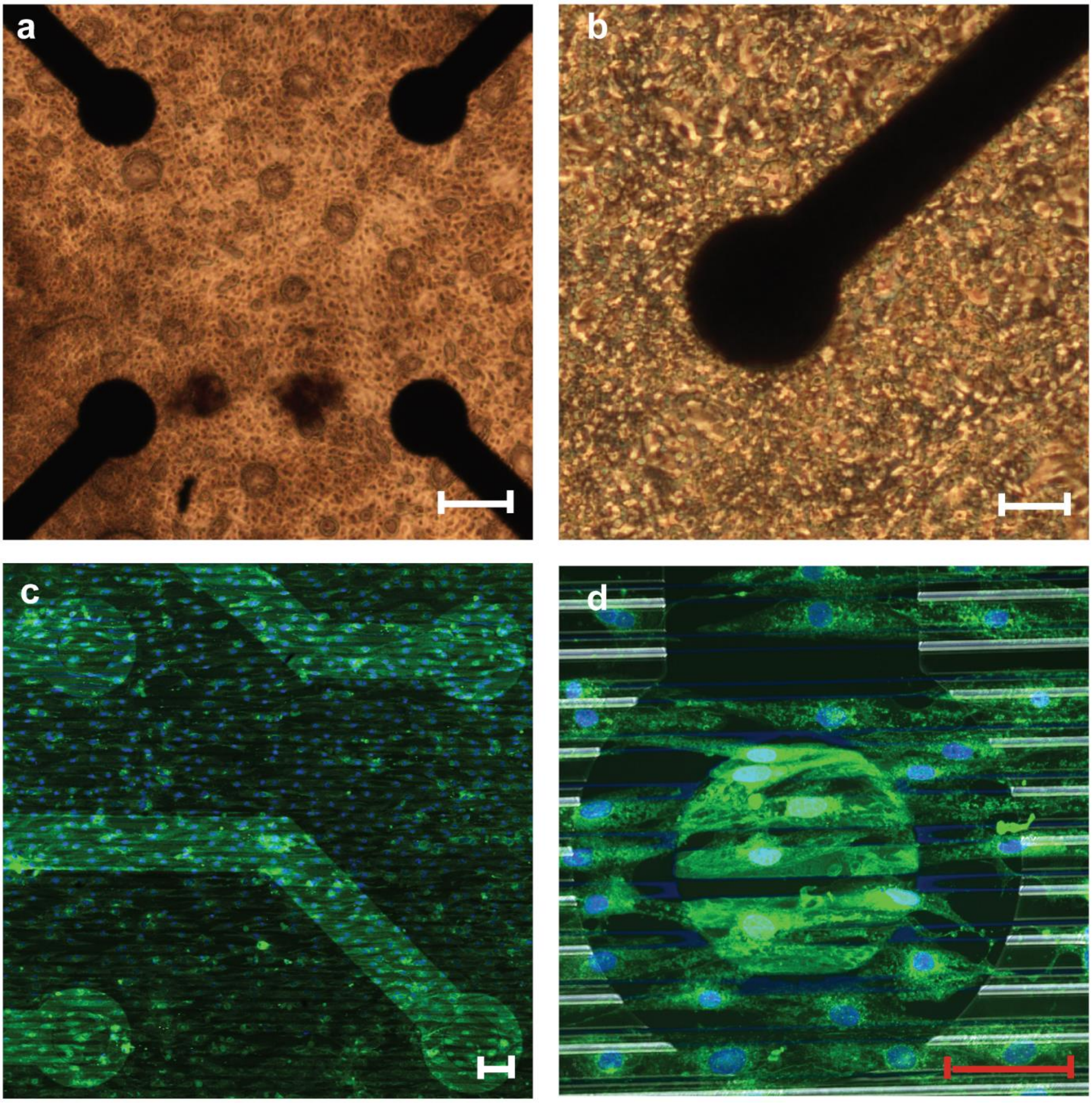

Supplementary Figure 3 | NRVM on pMEAs. (**a**)-(**b**) Bright-field images of NRVM cultured on standard (unpatterned) MEAs. (**c**)-(**d**) Immunofluorescence images of NRVM cultured on patterned MEA dishes stained for plasma membrane (green-wheatgerm agglutinin) and nuclear DNA (blue-DAPI). Scale bars, 100 μm (**a**); 50 μm (**b-d**).

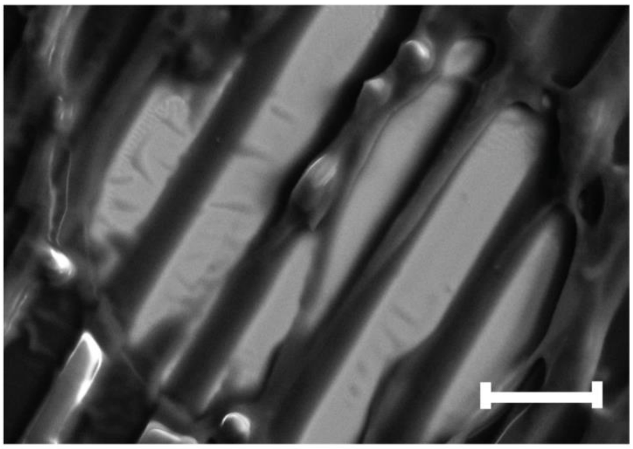

Supplementary Figure 4 | Scanning electron microscopy image, showing cells-electrode interface. Scale bar, 20 µm.

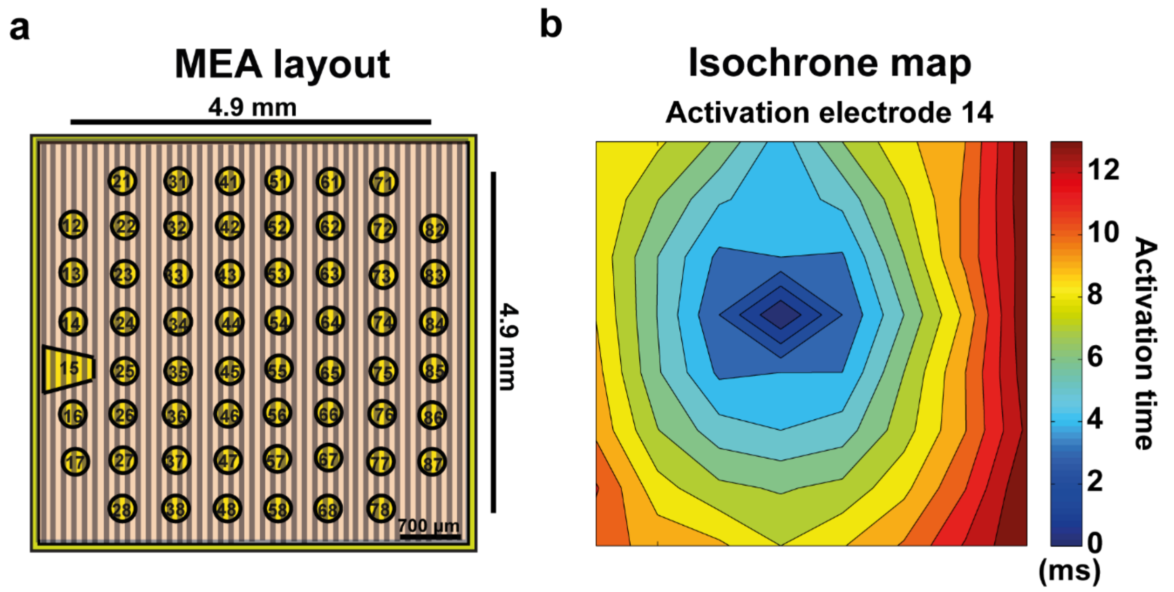

Supplementary Figure 5 | Off-the-shelf lifetime of micro-engineered MEAs. (**a**) Pattern layout and (**b**) corresponding isochrone map of an MEA that was micro-engineered 60 days before cell culturing.

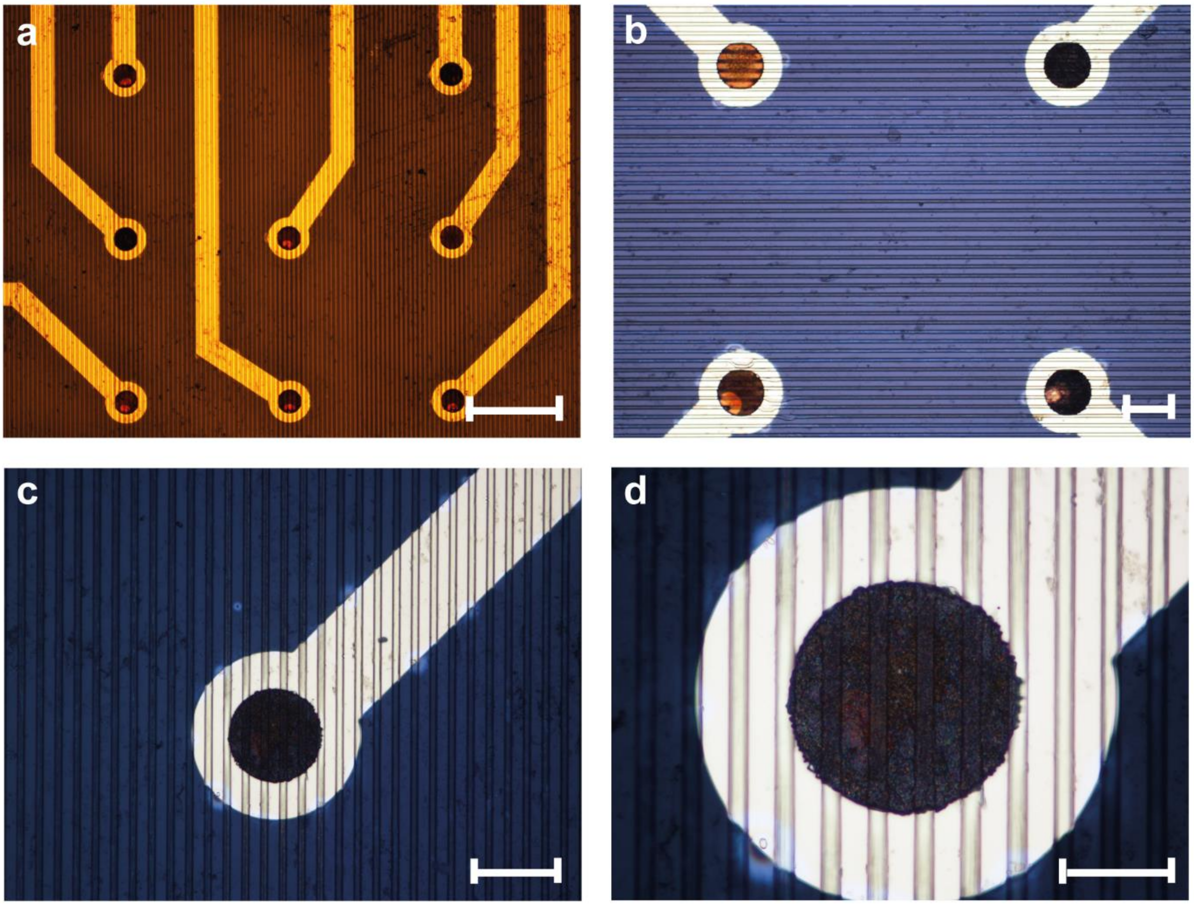

Supplementary Figure 6 | Micro-engineered MEAs after first use with cells. (**a**)-(**d**) Bright-field microscopy images of a micro-engineered MEA which was cleaned with water and dried after the first use with cells. Scale bars, 350 µm (**a**); 100 µm (**b**,**c**); 50 µm (**d**).

Supplementary Table 1 | Electrode impedance measurements (kOhms) at 1 kHz

	**Before processing[1]**	**After processing[2]**	**After first use[1]**
**Impedance (Z)**	64.03 ± 8.89	324.39 ± 115.83	112.17 ± 39.41
**Resistance (Re(Z))**	17.16 ± 1.73	158.85 ± 52.81	23.41 ± 11.47
**Reactance (Im(Z))**	61.62 ± 9.16	-278.67 ± 114.09	-109.40 ± 38.58

[1]Average value from 56 electrodes.
[2]Average value from 49 electrodes.
